# The Test-Retest Reliability of Non-Navigated Transcranial Magnetic Stimulation (TMS) Measures of Corticospinal Pathway Excitability in a Neurologically Intact Adult Population

**DOI:** 10.1007/s10548-026-01235-1

**Published:** 2026-07-27

**Authors:** Kathryn C. Collins, Allan B. Clark, Valerie M. Pomeroy, Niamh C. Kennedy

**Affiliations:** 1 School of Allied Health and Exercise Science, Bournemouth University, Bournemouth Gateway Building, St Pauls Lane, Bournemouth, BH8 8GP UK; 2 Norwich Medical School, University of East Anglia, Norwich, NR4 7TJ UK; 3 School of Health Sciences, University of East Anglia, Norwich Research Park, Norwich, NR4 7TJ UK; 4 School of Psychology, Ulster University, Cromore Rd, Coleraine, BT52 1SA UK; 5 Restoration Lead, National Institute for Health Research Health Technology Research Centre Brain Injury, Addenbrookes Hospital, Cambridge, CB2 0QQ UK; 6 Department of Clinical Neurosciences, University of Cambridge, Cambridge, CB2 0QQ UK

**Keywords:** Transcranial magnetic stimulation, Corticospinal tract, Reliability, Healthy adults

## Abstract

The test-retest reliability of Transcranial Magnetic Stimulation (TMS) measurements in healthy adults across the lifespan remains unclear. Determine the test-retest reliability and smallest detectable change (SDC) of motor evoked potential, (MEP), characteristics elicited by non-neuro-navigated Transcranial Magnetic Stimulation in neurologically intact adults. In two identical data collection sessions separated by five to seven days in a healthy adult sample was conducted. MEP characteristics assessed were motor threshold (MT) (rest and active conditions), amplitude (active), latency (MEP-L) (active conditions), silent period (SP) (active conditions), and recruitment curve (RC) (active conditions). Muscles investigated were bilateral biceps brachii (BB), extensor carpi radialis (ECR), and abductor pollicis brevis (APB). Test-retest reliability was calculated using the intra-class correlation coefficient (ICC) with corresponding 95% confidence intervals (CI) and limits of agreement (LOA), and the smallest detectable change was calculated. Acceptable reliability was set at a lower 95% CI of 0.70 or above for the ICC. In healthy adults (*n* = 51) none of the MEP characteristics were reliable. The lower end of the CI was below acceptable reliability (ICC < 0.7) for all MEP characteristics. Active MT of the non-dominant biceps was most reliable (e.g. ICC = 0.78[0.64–0.87]); whereas the RC demonstrated poor reliability for all muscles. The LOA had small mean differences between sessions with wide 95% LOA demonstrating measurement variability. The SDC were variable across muscles and MEP characteristics. In this study the findings indicate there is still limited evidence for acceptable reliability of TMS outcomes which are interpreted by the lower end of the 95% CI of the ICC.

**Supplementary Information:**

The online version contains supplementary material available at https://doi.org/10.1007/s10548-026-01235-1.

## Introduction

Transcranial magnetic stimulation (TMS) has been used with neurologically intact adults to develop knowledge of the corticospinal connection (Devanne et al. 2002; Pearce et al. 2000), to investigate neural plasticity e.g. (Pearce et al. 2000; Perez et al. 2004), and to induce virtual lesions to probe the contribution of specific brain areas to movement (Narayana et al. 2014; Vollmer et al. 2015). TMS studies in neurologically intact adults have been mainly focused on young adults, typically younger than forty years old (Carroll et al. 2001; Kamen 2004; Osnabruegge et al. 2023) with a lack of research in middle age and older adults. This may be problematic as the ageing process is associated with changes within the body’s systems, specifically the nervous system (Oliviero et al. 2006) as well as common diseases, for example hypertension, hypercholesterolemia, and diabetes.

The measurement of Motor Evoked Potentials (MEP) elements yields different findings in older adults compared to younger adults; however, the evidence is inconsistent. For example, previous research has exhibited the MEP amplitude in older adults to be smaller (McGinley et al. 2010; Oliviero et al. 2006); larger (Kossev et al. 2001); and no different to younger adults (Stevens-Lapsley et al. 2013). Despite the inconsistent findings, there is evidence of changes within the corticospinal pathway and its measurement with ageing. Examples of changes with age are a decrease in white matter, changes to tissue density, decrease in myelination and the number of myelinated neurons within the corticospinal pathway all of which may have an impact on TMS measurement and measurement reliability (Seidler et al. 2010; Salat et al. 2005). Understanding TMS measurement in neurologically intact adults within a range of ages, and with health conditions commonly seen in an ageing population (e.g. hypertension) can be more representative of the general population and contribute to normative data for comparison to middle age and older adults with neurological disease such as stroke as stroke survivors tend to be older (Xanthakis et al. 2014).

If inferences about the nervous system are drawn from TMS measurement, it is important that TMS measurement is reliable. The variability of TMS has been raised as a confounding factor to its potential use as a stable biomarker and test-retest reliability of the measurement is crucial to establishing TMS as a precise measurement parameter (Kanig et al. 2023). The test-retest reliability of TMS measures has been investigated in young healthy adults demonstrating moderate to good reliability e.g. (Carroll et al. 2001; Malcolm et al. 2006; Ngomo et al. 2012). Age-related changes in the brain and corticospinal pathway, and the changes in the MEP elements, may influence the test-retest reliability of TMS measures. There is a lack of robust TMS reliability research in middle age and older adults. There have been a few studies investigating the test-retest reliability of TMS measures in older adults (Christie et al. 2007; Houde et al. 2018; Schambra et al. 2015). However, these studies were limited to an assessment of hand muscles (Christie et al. 2007; Houde et al. 2018; Osnabruegge et al. 2023; Schambra et al. 2015 and limited to the assessment of MEP amplitude (Christie et al. 2007), motor threshold, and the recruitment curve (Schambra et al. 2015).

There is evidence that not all muscles respond equally to TMS, for example, the distal proximal gradient (Carson et al. 2013; Martin et al. 2006). It is expected that the reliability of TMS measurement will be different for different muscles. It is therefore essential to expand investigations beyond the hand muscles to the muscles of the forearm and upper arm muscles. This is important especially if TMS measures are to be applied to clinical populations such as stroke, where proximal muscles are key in recovery (Collins et al. 2024).

It has also been highlighted in previous research methodological issues with the interpretation of TMS reliability including lack of 95% confidence intervals (CI) for intraclass correlation coefficient (ICC) values when interpreting the findings, lack of powered samples and lack of a range of reliability measures such as smallest detectable change (SDC) (Beaulieu et al. 2017; Osnabruegge et al. 2023; Schambra et al. 2015).

The study reported here addressed many of these methodological concerns by investigating test-retest reliability: of a range of upper limb muscles, along the distal-proximal axis, in adults (including middle age and older adults) without neurological conditions with a sample size determined by a power calculation, and using ICCs with associated 95% CIs, limits of agreement (LOA), and determination of measurement error (smallest detectable change) (Koo and Li 2016), and dispersion of data around the mean using the coefficient of variation (CV).

## Aim

The aim of the study was to determine the test-retest reliability of MEP characteristics, elicited using non-neuro-navigated TMS in neurologically intact adults.

## Materials and Methods

### Design and Ethics

This study uses a prospective correlational test-retest reliability study design. The test-retest reliability of TMS measures of the corticospinal pathway was assessed over two identical TMS sessions separated by 5–7 days (Julkunen et al. 2009; Liu and Au-Yeung 2014).

Ethical approval was obtained from University Faculty of Medicine and Health Ethics (ref 2013 − 1420) for this study and the study was conducted in accordance with the Declaration of Helsinki. Participant testing was conducted at the University Exercise Laboratory.

### Participants

The estimated sample size was based on a power calculation. Assuming an intra-class correlation coefficient (ICC) of 0.80, then 51 neurologically intact participants would allow the estimation of the ICC with a 95% confidence interval (CI) of 0.7 to 0.9. This power calculation is based on published data for healthy adults (Beaulieu et al. 2017).

The inclusion criteria were aged 18 + years; reported that they did not have a diagnosis of a neurological disease; and had no contraindications to TMS as assessed by screening questionnaire (Rossi 2009). Participants were recruited via posters, email news bulletins, and community presentations.

### Procedure

The TMS methods are described in accordance with the guidelines on how to report on TMS methods and procedures (Chipchase et al. 2012). Procedures were identical in sessions one and two. The experimenter had extensive training in the use of TMS and was the same for both sessions. Participants were seated comfortably in an office chair with their forearms relaxed on a pillow placed on their lap. Arms were consistently positioned across sessions. Participants were instructed to attend to the limb being assessed, for example to maintain 20% of MCV for active trials as well as to attend to the limb during resting trials. Participants were not specifically instructed to relax any other part of their body during the testing procedures.

**Fig. 1:**
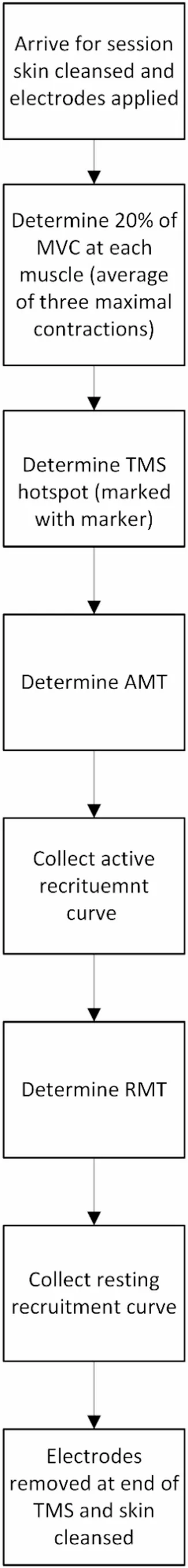
TMS procedures flow chart. Describes the TMS procedures at each TMS session which were identical

The muscles of investigation were the biceps brachii, extensor carpi radialis, and abductor pollicis brevis of both the dominant and non-dominant upper limbs. Figure 1 demonstrates the flow of the session of which the order for data collection was fixed. The muscles were assessed in the following order dominant biceps, non-dominant biceps, dominant ECR, non-dominant ECR, dominant APB, and non-dominant APB. The flow chart in Fig. 1 demonstrates the procedures that were repeated for each muscle. The active motor threshold was collected first as this mirrored a study the team completed in people after stroke in which the AMT was collected first.

Participants’ maximal voluntary contraction (MVC) of each target muscle bilaterally was assessed using a myometer (MTE Medical Research Limited), calculated as the mean of three maximum muscle contractions. Surface EMG electrodes were placed in parallel along the muscle fibres of the bilateral biceps brachii (BB) and extensor carpi radialis longus(ECR) (ConMed Cleartrace ECG surface electrodes 20 mm), cup electrodes (Nicolette ) placed over the abductor pollicis brevis (APB), and a ground electrode at the olecranon process. The EMG signal was sampled at 1000 Hz, amplified 1000x, band-pass filtered at 15–450 Hz, and saved for offline analysis. This process was standardized across all sessions. Single pulse monophasic TMS was delivered using a Magstim 200^2^ (Magstim Company Ltd) stimulator with a figure-of-8 coil (90 mm in diameter). The coil was placed so that the axis of intersection between the two loops was orientated at approximately 45^◦^ to the sagittal plane, to induce posterior to anterior current flow across the motor strip in the primary motor cortex (M1). At least 3 s was left between each TMS pulse. Once the hotspot for each muscle was established, the coil position was marked on the head and used throughout the experiment.

The hot spot was determined for each muscle individually at each session e.g. individual hot spot for dominant biceps, non-dominant biceps, dominant ECR, non-dominant ECR etc. (Carroll et al. 2001). The hot spot was the contralateral hemisphere to the muscle/limb of investigation such as the right hemisphere for the left arm and hand muscles, and the left hemisphere for the right arm and hand muscles. When the hotspot was identified for the muscle this was marked on the patient’s scalp with a marker. The active motor threshold, AMT, was determined while participants maintained 20% MVC via visual feedback from the myometer (Rossini and Rossi 2007). AMT was established when half of the successive pulses (5 in 10) produced an MEP > 200 µv (Rossini and Rossi 2007). The process for determining the AMT was repeated for each individual muscle. Resting motor threshold (RMT) was established as the lowest stimulation intensity at which MEPs with peak-to-peak amplitude of approximately 50 µv were evoked in at least 5 out of 10 MEPs, at that hotspot location. When identified for the muscle this was marked on the patient’s scalp with the marker. The process for determining the RMT was repeated for each individual muscle.

An active recruitment curve was obtained during 20% MVC from 100% AMT to 130% AMT increasing in 10% increments (bilateral limbs). Five TMS pulses were delivered at each intensity (Massie and Malcolm 2013; Collins et al. 2024). This process was repeated for each muscle (BB, ECR, APB) in both upper limbs. A resting recruitment curve (dominant limb only) was obtained from 90% RMT to 130% RMT increasing in 10% increments: five TMS pulses at each increment (Massie and Malcolm 2013). A fixed order of measures was selected for practical reasons i.e., to streamline the process of data collection, which was time-consuming and tiring for the participants (Koski et al. 2007).

### Statistical Analysis

The EMG signals were sampled at 1000 Hz, amplified 1000x, band-pass filtered at 15–450 Hz, and saved for offline analysis (Digitimer Ltd, Hertfordshire, UK), the CED Micro 1401 (Cambridge Electronic Design Limited, Cambridge UK), and the Neurolog System (Digitimer Ltd, Hertfordshire, UK).

The MEP measures recorded were active motor threshold, resting motor threshold, MEP amplitude, silent period (at 130% active motor threshold), MEP latency (at 120% active motor threshold), and recruitment curve slope. Following completion of data collection, all MEPs were visually assessed, then using a custom-made script, values were extracted for peak-to-peak MEP amplitude.

MEP latency was determined visually at 120% of AMT. A cursor was placed at the onset of the MEP defined as the first sustained crossing of the rectified EMG trace prior to the first MEP peak (Rossini et al. 2010; Daniel et al. 2015; Koski et al. 2007 b; Cacchio et al. 2009; Wasserman et al. 2002). The time from the stimulus to the cursor was the MEP latency in milliseconds (ms). The MEP latencies identified were comparable to previous research (Julkunen et al. 2009; Kossev et al. 2001).

Cortical Silent Period (CSP) was assessed visually from the baseline crossing after the 2nd MEP peak to visual return of EMG (Damron et al. 2008) at 130% AMT. Damron and colleagues (2008) identified that coefficient of variation was smaller for calculating the silent period visually versus mathematically and that the silent period at 130% AMT was homoscedastic thus least variable.

A second researcher assessed 10% of participants for agreement in MEP latency and silent period the two researcher’s were in agreement within 2 ms of each other in at least 80% of trials for both latency and silent period (Cacchio et al. 2011; Fisher et al. 2013). In instances in which the difference was greater than 2ms the researchers met, investigated the data and agreed upon the value.

The MEP amplitudes (following filtering and processing) were fitted using Boltzmann sigmoid function (Carroll et al. 2001; Carson et al. 2013; Massie and Malcolm 2013) which was MEP (S) = MEP_max_/1 + e^m(S50−S)^. and a linear function, which was y = a+bx (Massie and Malcolm 2013) to estimate component RC parameters. Of all the MEP trials collected, 8% of trials were not included in the analysis, this was spread across participants, due to an MEP not being present (130% of AMT was over 100% of stimulator output, or participant did not like high stimulation intensities), or there was electrical noise inhibiting the analysis of the MEP.

The data were tested for normality using a frequency histogram and exhibited a normal distribution. The test-retest reliability of TMS measures was assessed using different measures to give a holistic interpretation of the reliability. Firstly, the intra-class correlation coefficient (ICC) model [2,1] with associated 95% confidence intervals [95%CI] of individual measurement was used. ICC reflects the degree of correlation as well as agreement between ratings; how close the two scores are (Bruton et al., 2000, de Vet et al. 2006; Portney and Watkins 2009). The advantage of the ICC is that it supports generalizability in which the measured value is representative of the infinite distribution of possible values; thus the findings will be generalizable to the population (Portney and Watkins 2009). However, the ICC is limited in that it cannot determine absolute agreement only the percentage of variance (Bruton et al., 2000, Portney and Watkins 2009). Therefore, the Limits of Agreement (LOA) was also used. The LOA explores absolute reliability as well as any potential bias between the measurements (Bland and Altman 1986; Portney and Watkins 2009; Shrout and Fleiss 1979). Interpreting the ICC and LOA together can provide information about both the reliability and potential differences in TMS values between tests.

The ICC [2,1] model was used as TMS is used in individual prediction models and thus single measurement reliability is important, the formula used for the ICC [2,1] (two-way random effects, absolute agreement, single rater) is MS_R_-MS_E_ / MS_R_+(k-1)MS_E_+$\:\frac{k}{n}$ (MS_C_- MS_E)_. The formula used to determine the LOA was the mean difference between session 1 and session 2 plus or minus 1.96 times the standard deviation of the differences : LOA=mean difference ± 1.96(SD). The ICC model was interpreted with the associated 95% CI such that an ICC ≥ 0.70 is acceptable reliability, determined by the lower end of the 95% CI (Koo and Li 2016).

Additional reliability and variability measures explored were the smallest detectable change, and the coefficient of variation. The smallest detectable change is defined as the change in the individual’s score beyond measurement error (Atkinson and Nevill 1998). The SDC provides a value for the minimum change that needs to be observed, to be confident that the observed change is real and not, potentially, a product of measurement error (Geerinck et al. 2019). The smallest detectable change (SDC) was calculated using the formula: $\:1.96\:x\:\sqrt{2}\:x\:SEM\:$ (Beckerman et al. 2001). The formula used to calculate the SEM was SEM = SD*$\:\sqrt{1-r}$, where r is the estimated ICC value. The coefficient of variation (CV) was used to explore the dispersion of the data around the mean. This was calculated using pooled data (the mean of session 1 and session 1 as well as the average standards deviation of session 1 and session 2) using the formula CV=$\:\frac{SD}{Mean}$ x 100. (Atkinson and Nevill 1998).

## Results

**Fig. 2:**
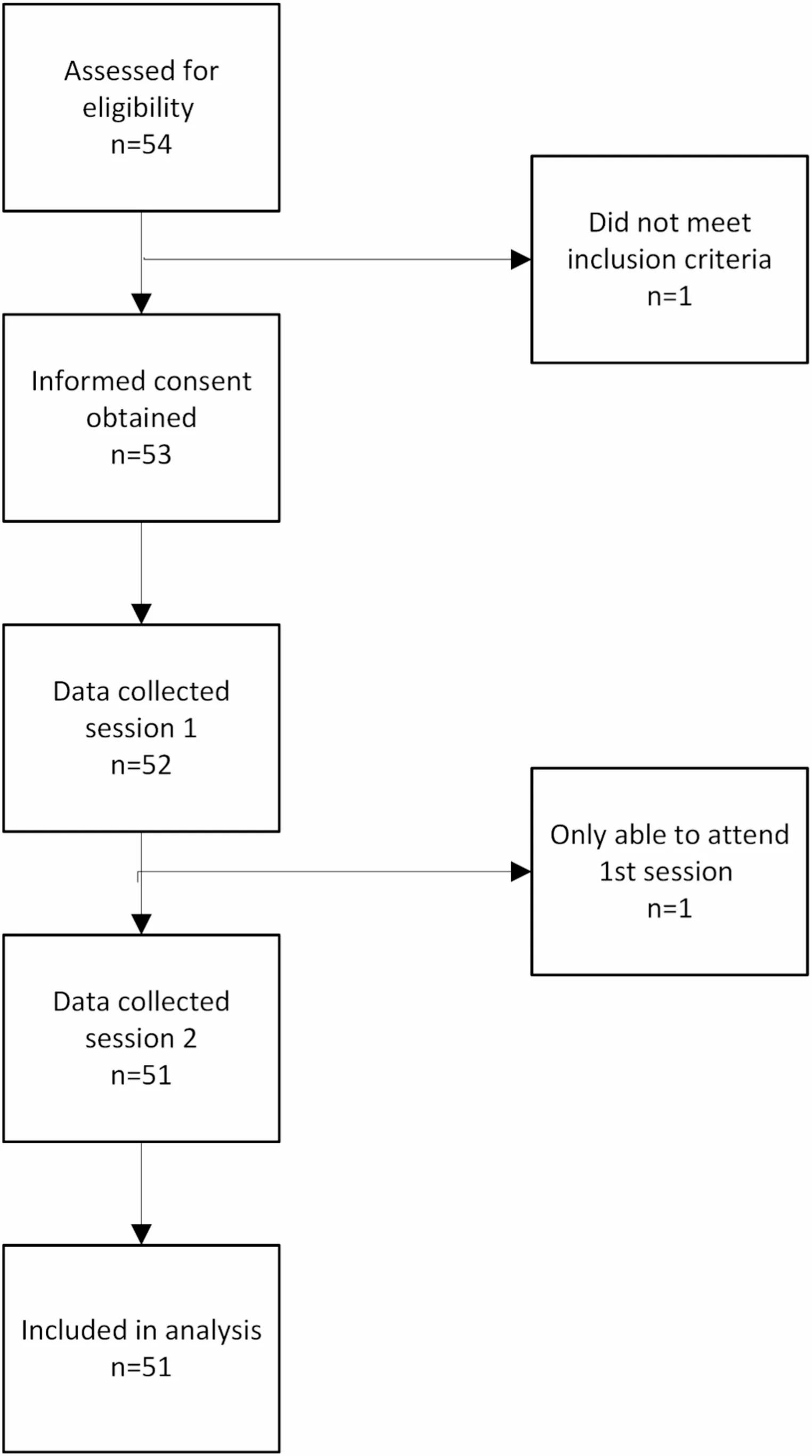
Recruitment flowchart. Describes the number of participants that were recruited and participated in the study

The required sample size of 51 healthy adults was achieved, see Fig. 2 for details. Participant characteristics are detailed in Table 1. There were no adverse events as a result of TMS. The mean and standard deviation of MEP characteristics are in Supplemental Table 1. The SDC should be interpreted in the context of whole group change, not individual change.

**Table 1:** Participant demographics. Describes the demographics, exercise level, and medication taken of the participants in the study *participating in exercise e.g., recreational activities, walking, running, or other physical activity

Demographic Element	Participant Description
Age in years (mean ± S D, range)	43.7 ± 16.4 years (range: 21–74)
Sex	Male *n* = 21 (41%) Female *n* = 30 (59%)
Hand dominance	Right *n* = 47 (92%) Left *n* = 4 (8%)
Participates in Exercise* ≤ 3x a week ˃ 3x a week	*n* = 40 (78%) *n* = 25 (63% of exercisers; 49% of participants) *n* = 15 (38% of exercisers; 29% of participants)
Consume caffeinated (within 24 h)	*n* = 44 (86%)
Medication	*n* = 20 (39%) Medication for Hypertension (*n* = 4; 20% of those taking medication; 8% of all participants) Contraception (*n* = 4; 20% of those taking medication; 8% of all participants) Asthma (*n* = 3; 15% of those taking medication; 6% of participants) SSRI (*n* = 2; 10% of those taking medication; 4% of all participants) Diabetic (*n* = 1; 5% of those taking medication; 2% of participants) Osteoarthritis (*n* = 2; 10% of those taking medication; 4% of all participants) Cholesterol (*n* = 1; 5% of those taking medication; 2% of participants) Stomach ulcer (*n* = 1; 5% of those taking medication; 2% of participants) Anemia (*n* = 1; 5% of those taking medication; 2% of participants) Thyroid hormone treatment (*n* = 1; 5% of those taking medication; 2% of participants)

### Curve Fitting

In healthy adults, the recruitment curve was fitted with a sigmoidal function in at least one of the sessions for 78% of participants for dominant BB, 80% of non-dominant BB, 76% of dominant ECR, 73% of non-dominant ECR, 71% of dominant APB, and 76% of participant’s non-dominant APB. Due to a proportion of participants lacking curve fitting with a sigmoidal function at both sessions, the recruitment curve was also fitted with a linear function.

Recruitment curves were fitted with linear functions in neurologically intact adults in at least one session for 100% of participants for dominant and non-dominant BB, dominant ECR, and dominant APB; 96% of non-dominant ECR; and 98% of non-dominant APB. For some participants 130% of motor threshold was above 100% of stimulator output, and others found the higher stimulation intensities uncomfortable thus these recruitment curves were fitted with less datapoints.

### Reliability ICC

The test-retest reliability of TMS measures in healthy adults was found to be variable both within and between measures and muscles (Table 2). The ICC [95% CI] ranged from; 0.00 [0.00, 0.25] for dominant APB active linear recruitment curve slope (Table 2); to 0.78 [0.64, 0.87] non-dominant BB AMT (Table 2). Of the estimated ICC values 7 of 48 measures fell within good reliability, 30 of 48 were moderate reliability and 11 of 48 were poor reliability (excluding the recruitment curve discussed below) (Koo and Li 2016). However, the lower end of the confidence interval fell below acceptable levels of reliability (0.7) for a majority of measures. Therefore, none of the MEP characteristics demonstrated acceptable test-retest reliability (lower 95% CI of 0.70 or greater). The slope of the recruitment curve demonstrated overall poor test-retest reliability (Table 2).

### Limits of Agreement

The LOA analysis demonstrated that mean differences between sessions were small, but the 95% CIs were wide, e.g., resting motor threshold (% of stimulator output) of the dominant ECR was + 0.14 (-10.98; 11.26). Overall, the Bland-Altman plots did not demonstrate systematic error between sessions. Thereby confirming variability in TMS measurement and lack of reliability when interpreted with the ICC.

### Smallest Detectable Change in MEP Characteristics

The SDCs of MEP characteristics are presented in Tables 2 and 3. For motor threshold, the SDCs ranged from 8.12% of stimulator output for AMT of non-dominant ECR to 11.10% for RMT of dominant ECR and APB. MEP-L SDCs ranged from 1.77ms for dominant BB to 3.43ms for non-dominant APB. The SP SDCs ranged from 41.46ms for dominant BB to 66.84ms for non-dominant APB. MEP amplitude SDCs ranged from 1.51µv for 100% AMT in dominant BB to 4.94µv for 110% AMT in non-dominant APB. Of the 24 MEP characteristics 15 were found to have a higher SDC for non-dominant than for dominant muscles. The SDC for the slope of the recruitment curve was not calculated as the reliability was poor for all muscles.

### Coefficient of Variation

The CV of MEP characteristics are presented in Tables 2 and 3. For motor threshold the CV ranged from 10.75% for non-dominant biceps RMT to 15.34% for the dominant ECR RMT. For MEP latency the CV ranged from 7.89% for the dominant ABP to 10.09% for non-dominant APB. For silent period CV ranged from 22.36% for dominant biceps to 34.01% for non-dominant ECR. The CV was not calculated for the slope of the recruitment curve as the reliability was poor for all muscles.

**Table 2:** Test-retest reliability, Limits of Agreement (LOA) Smallest Detectable Change (SDC) and coefficient of Variation (CV) of: motor threshold, motor evoked potential latency, silent period, and recruitment curve (RC). Demonstrates the test-rest reliability using the intraclass correlation coeffieicnt (ICC), limits of agreement, and smallest detectable change of TMS measures of the bilateral upper limb muscles for motor threshold (resting and active), MEP latency, silent period, and the recruitment curve (sigmoidal and linear)

SDC	Dominant UL ICC[95%CI]	Non-dominant UL ICC [95% CI]	Dominant UL			Non-dominant UL			
			Mean difference (95% LOA)	SDC	CV	Mean difference (95% LOA)	SDC		CV
*RMT*									
BB^A^	0.68 [0.49,0.80]	0.76 [0.60,0.86]	-0.37 (-12.84;12.11)	12.18	12.52%	-0.22 (-9.47; 9.03)	9.12	10.75%	
ECR^A^	0.71 [0.54,0.82]	0.69 [0.51,0.81]	-0.64 (-12.02;10.74)	11.10	15.34%	-0.20 (-11.03; 10.63)	10.60		13.73%
APB^A^	0.69 [0.52,0.81]	0.68 [0.50,0.81]	0.14(-10.98; 11.26)	11.10	14.67%	0.64 (-9.69; 10.98)	10.34		12.83%
*AMT*									
BB^A^	0.76 [0.61,0.85]	0.78 [0.64,0.87]	0.65 (-9.37; 10.66)	9.75	15.12%	0.61 (-9.03; 10.25)	9.35		15.13%
ECR^A^	0.59 [0.38, 0.74]	0.67 [0.49,0.80]	0.59 (-8.38; 9.55)	8.79	13.01%	0.26 (-8.07; 8.58)	8.12		12.74%
APB^A^	0.55 [0.32,0.71]	0.56 [0.34,0.72]	0.35 (-9.02; 9.72)	9.11	12.09%	0.92 (-7.85; 9.69)	8.62		11.30%
*MEP-L*									
BB^A^	0.59 [0.38, 0.74]	0.61 [0.41, 0.76]	-0.05 (-0.2.71; 2.60)	1.77	7.92%	-0.11 (-2.34; 2.12)	2.13		9.86%
ECR^A^	0.65 [0.46, 0.79]	0.56 [0.34, 0.72]	0.32 (-2.40; 3.03)	2.50	9.12%	0.44 (-2.24; 3.13)	2.56		8.56%
APB^A^	0.56 [0.35, 0.72]	0.70 [0.52, 0.82]	-0.34 (-4.75; 4.07)	3.13	7.89%	0.06 (-3.39; 3.51)	3.43		10.09%
*SP*									
BB^A^	0.61 [0.41, 0.76]	0.54 [0.31, 0.71]	-5.61 (-47.34; 36.13)	41.46	22.36%	-3.69 (-53.81; 46.42)	49.80		24.71%
ECR^A^	0.66 [0.47, 0.79]	0.75 [0.60, 0.85]	-0.83 (-47.73; 46.06)	45.97	28.63%	-1.40 (-52.76; 50.00)	50.38		34.01%
APB^A^	0.65 [0.42, 0.79]	0.63 [0.42, 0.77]	-11.08 (-61.98; 39.81)	52.56	24.28%	-1.35 (-69.66; 66.89)	66.84		29.91%
*RC-S*									
BB	0.04 [0.00, 0.45]^B^	0.02 [0.00, 0.60]^C^	0.06 (-0.06; 0.17)^B^	NT		0.03(-0.390;0.63)^C^	NT		
ECR	0.00 [0.00, 0.36]^D^	0.27 [0.00, 0.52]^E^	-0.07 (-0.37; 0.24)^D^	NT		0.03 (0.00; 0.31)^E^	NT		
APB	0.30 [0.00, 0.68]^B^	0.37 [0.00, 0.72]^F^	-0.05 (-0.25; 0.13)^B^	NT		0.40 (-0.31;0.06)^F^	NT		
*RC-L*									
BB^G^	0.50 [0.26, 0.68]	0.01 [0, 0.19]	-0.01 (-0.09; 0.08)	NT		0.01 (-0.07; 0.08)	NT		
ECR^H^	0.23 [0.00, 0.48]	0.05 [0, 0.21]	-0.02 (-0.25; 0.22)	NT		0.01 (-0.07; 0.09)	NT		
APB^I^	0.00 [0.00, 0.25]	0.02 [0, 0.18]	0.05 (-0.35; 0.24)	NT		0.01 (-0.14; 0.15)	NT		

UL= upper limb; BB= biceps brachii; ECR= extensor carpi radialis; APB= abductor pollicis brevis; RMT= resting motor threshold, measured at % of stimulator output; AMT= active motor threshold, measured as % of stimulator output; MEP-L= motor evoked potential latency, measured in milliseconds; SP= silent period, measured in milliseconds; RC-S= active recruitment curve fitted with sigmoidal function; RC-L recruitment curve fitted with a linear function; ICC= intraclass correlation coefficient (ICC model 2,1); LOA = limits of agreement; SDC= smallest detectable change; CV= coefficient of variation; NT = not tested due to poor reliability of the recruitment curve slope; ^A^= 51 participants; ^b^= 16 participants; ^C^= 15 participants; ^D^= 11 participants; ^E^= 12 participants; ^F^= 13 participants; ^G^= 31 participants; ^H^=23 participants; ^I^= 29 participants

**Table 3:** Test-retest reliability, Limits of Agreement (LOA), Smallest Detectable Change (SDC), and Coefficient of Variation (CV) of MEP amplitude at different stimulation intensities. Demonstrates the test-retest reliability using the Intraclass Correlation Coefficient (ICC), limits of agreement smallest detectable change, and coefficient of variation of MEP amplitude of upper limb muscles at different stimulation intensities from 100% of active motor threshold to 130% of active motor threshold

	Dominant UL	Non-dominant UL	Dominant UL				Non-dominant UL				
	ICC [95% CI]	ICC [95% CI]	Mean difference (95% LOA)	SDC		CV	Mean difference (95% LOA)	SDC			CV
*BB*											
100% AMT	0.43 [0.17, 0.63]	0.54 [0.31, 0.71]	-0.09 (-1.61; 1.43)	1.51	70.24		0.14 (-1.60; 1.88)	1.67	77.63		
110% AMT	0.47 [0.22, 0.65]	0.53 [0.30, 0.70]	-0.32 (-2.17; 1.53)	1.53		55.07	0.21 (-2.07; 2.49)	2.96	72.19		
120% AMT	0.45 [0.21, 0.65]	0.63 [0.43, 0.77]	-0.39 (-2.77; 1.98)	2.10		56.67	0.26 (-2.14; 2.67)	2.36	69.31		
130% AMT	0.50 [0.27, 0.68]	0.49 [0.26, 0.67]	-0.40 (-3.09; 2.29)	2.39		56.00	0.42 (-3.02; 3.87)	3.37	70.75		
*ECR*											
100% AMT	0.64 [0.45, 0.78]	0.51 [0.28, 0.69]	0.15 (-3.02; 3.32)	3.11		91.22	-0.14 (-1.89; 1.62)	1.71		59.06	
110% AMT	0.75 [0.60, 0.85]	0.51 [0.27, 0.69]	0.12 (-2.82; 3.07)	2.87		83.30	0.07 (-2.20; 2.06)	2.08			56.80
120% AMT	0.76 [0.62, 0.86]	0.56 [0.33, 072]	0.07 (-2.88; 3.03)	2.88		74.78	0.05 (-2.06; 2.17)	2.06			51.98
130% AMT	0.76 [0.62, 0.86]	0.48 [0.23, 0.66]	0.03 (-2.86; 2.93)	2.84		65.83	0.03 (-2.41; 2.48)	2.38			50.21
*APB*											
100% AMT	0.13 [0.00, 0.38]	0.46 [0.21, 0.65]	0.38 (-2.21; 2.97)	2.38		73.49	0.30 (-3.18; 3.79)	3.46			94.69
110% AMT	0.44 [0.19, 0.64]	0.28 [0.01, 0.51]	0.16 (-2.78; 3.09)	2.91		75.89	0.57 (-4.45; 5.58)	4.94			84.31
120% AMT	0.33 [0.05, 0.55]	0.51 [0.27, 0.69]	-0.00 (-4.01; 4.10)	3.90		67.85	0.68 (-3.93; 5.28)	4.60			74.65
130% AMT	0.31 [0.03, 0.54]	0.55 [0.32, 0.72]	-0.17 (-4.50; 4.16)	4.24		60.93	0.41 (-4.31; 5.14)	4.67			68.02

MEP= motor evoked potential, measured in µv; AMT = active motor threshold, % of stimulator output; BB = biceps brachii muscle; ECR = extensor carpi radialis muscle; APB = abductor pollicis brevis muscle; ICC= intraclass correlation coefficient (model 2,1); LOA = limits of agreement, SDC = smallest detectable change; CV= coefficient of variation *n* = 51 participants

## Discussion

In this present study we characterized the reliability of frequently used TMS measures in neurologically intact adults from a range of ages (21 to 74 years of age) in a range of upper limb muscles across two test-retest sessions. Although individual scores and estimated ICC values showed moderate reliability, when interpreted with the CI and other measures it presents a much less reliable picture. This study found that most TMS measures were not reliable due to the lower end of 95% CI of the ICC being below 0.7. Despite many of the estimated ICC values being moderate or good reliability, they must be interpreted in conjunction with 95% CI values. TMS measurement is variable as indicated by the wide LOA, large SDC and CV for MEP characteristics.

When comparing the reliability identified in this study, some measures are similar to previous research such as the reliability of the APB motor threshold (Sollmann et al. 2013). Other ICC values of this study are lower compared to previous research for the MEP amplitude, silent period, and MEP latency (Carroll et al. 2001; Christie et al. 2007; Li and Au-Yeung 2014; Ngomo et al. 2012; Schambra et al. 2015; Koski et al. 2007). Studies explored different muscles, mainly conducted in young healthy adults, used different TMS methodologies, and different analysis methods contributing to heterogeneity in the research. Highlighting a need for research to expand into broader age ranges and development of a robust TMS methodology that can be used across studies. The CV was large for all measures indicating that MEP characteristics are variable relative to the mean. This was supported by the wide limits of agreement suggesting the absolute differences between the measurements were large around a small mean difference between the two sessions, further indicating variability. A benefit of using the CV is that it is a dimensionless statistic thus allowing comparison between measurements despite how they are measured.

The SDC identified in this study was large for all measures. The SDC identified was larger when compared to previous research such as SDC of resting motor threshold (Schambra et al. 2015) and a synthesis of SDC measures in a systematic review (Beaulieu et al. 2017). The SDC can be used in clinical practice for clinicians to identify if there has been a change in corticospinal pathway excitability that is greater than measurement error. For example, a systematic review by Veldema and colleagues (2021) synthesised the change in motor threshold in people after stroke following upper limb rehabilitation. The change in motor threshold ranged from a change of 1% of stimulator output to 13% of stimulator output with a majority below 10% of stimulator output (Veldema et al. 2021). It is challenging to compare SDC in healthy people to those after stroke, however the SDC in this for motor threshold ranged from 9% stimulator output to 12% stimulator output at the higher end of the observed change. Thus, it is impractical for TMS to be used at an individual level to assess neuroplasticity, but may be more appropriate to be used at a group level.

The reliability of the individual muscles varied which is in line with earlier work (Carson et al. 2013; Kamen 2004; Malcolm et al. 2006). This finding is not surprising as there is evidence that the muscles of the upper limb respond differently to brain stimulation and thus the reliability may be different (Martin et al. 2006). The majority of previous test-retest studies have focused on distal muscles (Christie et al. 2007; Schambra et al. 2015; Sollmann et al. 2013) with only a handful looking at forearm muscles (Malcolm et al. 2006).

Other factors can impact on the excitability of the corticospinal pathway for example SSRI medications and female hormones. There is evidence that SSRI medication can increase the excitability of the corticospinal pathway (Khedr et al. 2020) and may impact on the reliability of the measurements, two participants were taking SSRI and thus this is likely to have had little influence on reliability. Additionally, it has been demonstrated that women have a greater variability in response to TMS compared to men (Pitcher et al., 2003, Smith et al., 2011). This is thought to be due by female hormones during the menstrual cycle and menopause such as progesterone (Smith et al., 2002, Smith et al. 1999; Wassermann 2002). Progesterone is associated with GABA which acts as an inhibitory neurotransmitter during the menstrual cycle phase, when progesterone (Smith et al. 1999) is high which may have an impact on the reliability of the measurement if the two sessions were completed at different phases of the menstrual cycle.

The reliability of the recruitment curve was poor for all muscles and limbs. Potential reasons for the poor reliability is the reliability of the MEP amplitude and the number of data points within the recruitment curve, and challenges to curve fitting. Firstly, the reliability of MEP amplitude in the present study was below acceptable reliability for most muscles. The MEP amplitude is the basis for plotting the recruitment curve, therefore if the amplitude is variable then subsequently the reliability of recruitment curve may also be variable and have poor reliability. Secondly, 130% of motor threshold was above 100% of stimulator output for some participants, thus there were no data points for this stimulation intensity, the curve was plotted at 10% increments of stimulator output. Some participants found the high stimulation intensities uncomfortable, leading to fewer data points within some recruitment curves. Having fewer data points within the recruitment curve could have contributed to challenges with curve fitting and the poor reliability. There were challenges with recruitment curve fitting using a sigmoidal function, having more success fitting the recruitment curve with a linear function. This is similar to previous studies that also had challenges with curve fitting with a sigmoidal function (Massie and Malcolm 2013; Ray et al. 2002; Schambra et al. 2015).

There are challenges to interpretation of the slope of the recruitment curve when using the sigmoid function due to small differences in how the slope is characterised within the equation as well as different equations can be used such as the inverse of the slope. The linear function may be influenced by data collection methods. However, Massie and Malcolm (2013) identified that the peak slope of a sigmoid function was correlated to the slope of the linear function (Massie and Macolm 2013). An advantage to a linear function is that it is less mathematically complex, and requires fewer data points than a sigmoid function. Thus, in the case of this study the linear function was easier to fit to the data in instances where the data at 130% was missing due to being over 100% of stimulator output or due to participant preference at higher stimulation intensities. There is not an agreed best method for recruitment curve fitting, future research should explore a standardised approach which would ease comparisons across studies.

Many ICC models can be used to determine reliability, the model chosen can influence the reliability and should be considered when comparing between studies. The ICC model [2,k] which is often used is the reliability of the mean of observations; ICC model [2,1] is the reliability of individual observations and more accurate in this study (Portney and Watkins 2009), as TMS is being used in individual prediction models. For example, model ICC [2,1] can be influenced by systematic differences inherent in measurement error, whereas model ICC [2,k] does not account for systematic differences (De Vet et al. 2006). Therefore, the ICC values resulting from model ICC [2,1] will be lower than those of ICC [2,k] (De Vet et al. 2006; Portney and Watkins 2009). The ICC model used in the present study was ICC [2,1] which demonstrated lower ICC values than research that utilised other ICC models e.g. ICC [2,k] (Malcolm et al. 2006; Schambra et al. 2015). It may be the ICC model used is contributing to the difference in ICC values between studies, not the agreement between tests. Furthermore, many studies did not report the ICC with the associated 95% CI. It is not possible to determine the variability in measure in those studies as well as if the lower end of the 95% CI falls within acceptable reliability.

### Strengths and Limitations

This study was adequately powered to investigate the research question, an issue that has been highlighted in the literature (Beaulieu et al. 2017). The participants had a higher average age than the majority of other TMS reliability studies e.g. (Osnabruegge et al. 2023), with an age range more representative of the adult life span 21–74. This study implemented the 5 key optimizations as suggested by Osnabruegge and colleagues (2023) including at least 5 stimuli, 110% of RMT, figure of 8 coil and monophasic waveforms. TMS measures were collected during resting conditions and during background muscle contraction. This is a strength as an application into clinical populations (such as stroke) may necessitate the collection of responses during background activity due to higher motor thresholds.

The TMS methods used within this study may result in the findings not being comparable to other studies that utilized different methodologies. TMS was non-navigated, there is variation across the research base on the use of neuro-navigated TMS. TMS data was collected during background muscle contraction which may not be comparable to earlier studies in neurologically intact adults in which TMS data is collected at rest. Resting measures were only collected in the dominant limb rather than bilateral limbs. The amount of arm use before the TMS session was not restricted nor was it recorded. There could have been varying levels of corticospinal pathway excitability prior to each session. The recruitment curve was not able to be fitted for all participants, thus the analysis of test-retest reliability of the slope of the recruitment curve was underpowered. The methodology used a standardised protocol for TMS data collection which has benefits in minimizing procedural variability and ease of replication. However, employing a random order of TMS data collection could have contributed to reduced order effects and habituation, as well as reduce within session variability which may have contributed to the reliability findings in this study. Five TMS pulses were given at each intensity level, this could be a limitation as evidence since the study was conducted suggests more TMS pulses at data collection point, at least 20. With an increasing number of TMS pulses intra-session variability improved (Biabani et al. 2018). Using 5 TMS pulses may have contributed to variability within the data and poorer reliability.

This study did not use neuro-navigated TMS, this decision was made to align it with its potential use in a clinical population where access to gold-standard laboratories with neuro-navigation is not always possible. Consequently, if TMS is to be used widely in clinical neurorehabilitation, then TMS used without neuro-navigation also needs to demonstrate acceptable test-retest reliability (Collins et al. 2024). There has also been a mix of success with neuro-navigation improving TMS measure reliability (Schambra et al. 2015; Tedesco Triccas et al. 2018) but other research suggests that the use of neuro-navigation or not has less of an impact than other sources of MEP variability (Julkunen et al. 2009; Osnabruegge et al. 2023).

## Conclusions

This study determined that, within the population assessed, the test-retest reliability of TMS measures is variable, as well as demonstrating wide 95% CI, 95% LOA, and large SDC suggesting imprecision in TMS measurement.

## Supplementary Information

Below is the link to the electronic supplementary material.

## Data Availability

No datasets were generated or analysed during the current study.
