# Supplementary material for: The Test-Retest Reliability of Non-Navigated Transcranial Magnetic Stimulation (TMS) Measures of Corticospinal Pathway Excitability in a Neurologically Intact Adult Population
Source: Brain Topogr. 2026 Jul 27;39(5):85. doi: 10.1007/s10548-026-01235-1 (PMC13407758; doi:10.1007/s10548-026-01235-1)
Supplement: Supplementary file 1 — Supplementary Material 1 [file 10548_2026_1235_MOESM1_ESM.docx]

**The test-retest reliability of non-navigated transcranial magnetic stimulation (TMS) measures of corticospinal pathway excitability in a healthy population across the adult lifespan**

Kathryn C Collins PhD ^a ,^ Allan B Clark, PhD ^c^ Valerie M Pomeroy, PhD ^d,e^ and Niamh C Kennedy, PhD ^b^

Journal: Neurological Sciences

Corresponding Author: Kathryn C Collins; Faculty of Health and Social Sciences, Bournemouth University, Bournemouth Gateway Building, St Pauls Lane, Bournemouth, BH8 8GP, UK; [kcollins@bournemouth.ac.uk](mailto:kcollins@bournemouth.ac.uk)

**Supplemental Material**

**Supplemental Table 1. Mean (standard deviation) of MEP characteristics for adults for resting motor threshold (% stimulator output), active motor threshold (% stimulator output), motor evoked potential latency (ms) and silent period (ms)**

| **Muscle** | **RMT Session 1** | **RMT Session 2** | **AMT Session 1** | **AMT Session 2** | **MEP-L Session 1** | **MEP- L Session 2** | **SP Session 1** | **SP Session 2** | **RC Session 1** | **RC Session 2** |
| --- | --- | --- | --- | --- | --- | --- | --- | --- | --- | --- |
| **Biceps** | | | | | | | | | | |
| D | 62±8.47 | 62±7.06 | 48±7.26 | 47±7.10 | 12.48±1.01 | 12.65±0.98 | 103.47±25.04 | 110.74±22.85 | 0.12±0.11 | 0.10±0.08 |
| ND | 62±6.84 | 63±6.60 | 48±7.32 | 47±7.06 | 12.39±1.30 | 12.56±1.16 | 105.27±25.61 | 109.13±27.36 | 0.23±0.26 | 0.11±0.05 |
| **ECR** | | | | | | | | | | |
| D | 48 ±6.87 | 49 ±8.01 | 38 ±4.86 | 38 ±5.03 | 16.48±1.52 | 16.40±1.48 | 98.03±28.21 | 100.61±28.66 | 0.21±0.38 | 0.09±0.07 |
| ND | 50.00±6.83 | 50.00±6.90 | 40.00±4.92 | 40.00±5.27 | 16.38±1.57 | 16.08±1.21 | 107.45±35.43 | 106.33±37.27 | 0.14±0.07 | 0.12±0.06 |
| **APB** | | | | | | | | | | |
| D | 49.00±7.07 | 49.00±7.31 | 41.00±5.24 | 40.00±4.55 | 22.93±1.87 | 22.59±1.72 | 126.56±31.05 | 137.48±33.05 | 0.17±0.12^A^ | 0.19±0.20^A^ |
| ND | 51.00±6.77 | 50.00±6.19 | 42.00±4.95 | 41.00±4.43 | 22.31±2.22 | 22.49±2.30 | 133.29±40.03 | 131.79±39.25 | 0.17±0.16^B^ | 0.14±0.12^B^ |

**Supplemental Table 1.** Demonstrates the mean and standard deviation of TMS measures (motor threshold (active and resting), MEP latency, silent period, and recruitment curve (sigmoidal)

S1 = session 1; S2 = session 2; D= dominant limb; ND= ND limb; RMT = resting motor threshold, measured in % of stimulator output; BB = biceps brachii muscle; ECR = extensor carpi radialis muscle; APB = abductor pollicis brevis muscle; AMT = active motor threshold, measured in % of stimulator output; MEP-L = motor evoked potential latency, measured in milliseconds; SP = silent period, measured in milliseconds, RC= recruitment curve fitted with a sigmoidal function; n= 51 participants unless stated otherwise; ^A^= 16 participants; ^B^= 15 participants
